# Convergence in fertility of South Africans and Mozambicans in rural South Africa, 1993–2009

**DOI:** 10.3402/gha.v6i0.19236

**Published:** 2013-01-24

**Authors:** Jill Williams, Latifat Ibisomi, Benn Sartorius, Kathleen Kahn, Mark Collinson, Stephen Tollman, Michel Garenne

**Affiliations:** 1 Institute of Behavioral Science, Population Program, University of Colorado Boulder, Boulder, CO, USA; 2 MRC/Wits Rural Public Health and Health Transitions Research Unit, School of Public Health, Faculty of Health Sciences, University of the Witwatersrand, Johannesburg, South Africa; 3 Demography and Population Studies Programme, School of Social Sciences, Faculty of Humanities, University of the Witwatersrand, Johannesburg, South Africa; 4 School of Public Health, Faculty of Health Sciences, University of the Witwatersrand, Johannesburg; 5 Centre for Global Health Research, Umeå University, Umeå, Sweden; 6 INDEPTH Network, Accra, Ghana; 7 Epidémiologie des Maladies Emergentes, Institut Pasteur, Paris, France; 8 Institut de Recherche pour le Développement (IRD), Unite Mixte Internationale (UMI) Résiliences, Paris, France

**Keywords:** fertility decline, education, adolescent fertility, birth intervals, labour force participation, contraception, socio-economic development, refugees, adaptation, Agincourt health and socio-demographic surveillance site

## Abstract

**Background:**

Although there are significant numbers of people displaced by war in Africa, very little is known about long-term changes in the fertility of refugees. Refugees of the Mozambican civil war (1977–1992) settled in many neighbouring countries, including South Africa. A large number of Mozambican refugees settled within the Agincourt sub-district, underpinned by a Health and Socio-demographic Surveillance Site (AHDSS), established in 1992, and have remained there. The AHDSS data provide a unique opportunity to study changes in fertility over time and the role that the fertility of self-settled refugee populations plays in the overall fertility level of the host community, a highly relevant factor in many areas of sub-Saharan Africa.

**Objectives:**

To examine the change in fertility of former Mozambican self-settled refugees over a period of 16 years and to compare the overall fertility and fertility patterns of Mozambicans to host South Africans.

**Methods:**

Prospective data from the AHDSS on births from 1993 to 2009 were used to compare fertility trends and patterns and to examine socio-economic factors that may be associated with fertility change.

**Results:**

There has been a sharp decline in fertility in the Mozambican population and convergence in fertility patterns of Mozambican and local South African women. The convergence of fertility patterns coincides with a convergence in other socio-economic factors.

**Conclusion:**

The fertility of Mozambicans has decreased significantly and Mozambicans are adopting the childbearing patterns of South African women. The decline in Mozambican fertility has occurred alongside socio-economic gains. There remains, however, high unemployment and endemic poverty in the area and fertility is not likely to decrease further without increased delivery of family planning to adolescents and increased education and job opportunities for women.

Africa is home to about a fifth of the world's refugees, most of whom have been victims of forced migration (1).‡ However, little is known about the long-term impact of refugee status on fertility rates. Most studies of migration and fertility in Africa have focussed on examining the impact of rural to urban migration on fertility or, less common, the impact of circular migration on fertility in rural populations (2–4).

War and resettlement can place both upward and downward pressure on fertility in the short term. Upward pressure may come from the desire to replace those lost in war, while downward pressure on fertility may come from the disruption of life and relationships caused by war (5). Studies of these effects over the short term find that many factors – including social characteristics of people prior to war – determine fertility levels in the short- and medium-term after war (5, 6). Biological factors such as sub-fecundity caused by malnutrition can also play a role in suppressing refugee fertility in the short term. Studies on forced migration and resettlement suggest that fertility of refugees in the long run is influenced by the same social and demographic factors that impact on fertility for everyone, such as education, age, socio-economic status, and urban or rural residence (5).

However, most studies on refugee fertility are conducted in refugee camps and the situation may differ for refugees not living in camps. Populations that settle in host countries without residing in camps are likely to be different from those in refugee camps since they are not served directly by aid programs. Many studies of the fertility of self-settled refugees exist in developed countries with vital registration systems. However, studies of self-settled refugee populations in Africa where vital registration systems are lacking are rare. Prospective data from the Agincourt sub-district in Mpumalanga Province in rural northeast South Africa provide an opportunity to examine the change in fertility of self-settled Mozambican refugees over a period of 16 years (1993–2009) and to examine their impact on overall fertility levels in the area. Earlier research using data from the Agincourt health and socio-demographic surveillance site (AHDSS) found that Mozambican refugees in Agincourt contributed to a noticeable increase in the average number of children borne (total fertility rate – TFR) by women in the 1980s measured retrospectively through birth histories (7). Subsequently, the TFR for all of Agincourt has dropped from 3.7 in 1993 to a low of 2.3 in 2002 and has hovered around 2.5 since then. This fertility decline is similar to that across rural South Africa during the same period (6, 7).

Fertility decline in South Africa generally is attributed in part to the widespread use of modern contraceptives. A national family planning programme was started in 1974 in large part due to an ideological response by the *apartheid* regime to the spectre of rapid population growth among the African population. The programme provided free modern contraceptives in public health clinics, including oral and injectable contraceptives (6, 7). The 1998 Demographic and Health Survey found that 51.2% of sexually active African women in rural areas used some form of modern contraception (8). In 2003 this number increased to 61% (9).

Despite the observed decrease in fertility in the AHDSS since the early 1990s, little is known about the fertility of Mozambican women over time in Agincourt – if, when, and how fast their fertility decreased since the early 1990s. In this study, we examine changes in fertility levels and patterns over time through a comparative analysis between the two main population groups in Agincourt – South Africans and Mozambicans. We examine (1) TFRs, (2) age specific fertility rates (ASFR), (3) timing of first and second births, and (4) trends in selected socio-economic characteristics likely to influence changes in fertility of Mozambican women in Agincourt.

## Methods

We use prospective longitudinal data on births in the AHDSS in Agincourt to examine the fertility of Mozambican women and compare it to that of African South Africans in the same area. The AHDSS covers much of what is now the Agincourt sub-district but was previously part of an *apartheid* Bantustan (or ‘homeland’), Gazankulu, where African South Africans were resettled as part of the apartheid regimes strategy of ‘separate development’ (10). Most of the people, both South Africans and Mozambicans, in this area belong to the Shangaan Speaking.

Agincourt itself is only about 40 km west of the southern Mozambique border. About a third of the population living in the area covered by the AHDSS are Mozambican, most having entered the country as refugees in the early to mid-1980s during the Mozambican civil war between 1977 and 1992 (11). Despite voluntary repatriation programmes in 1994, a large proportion of refugees elected to stay in the area. Therefore, the AHDSS arguably contains the largest population of self-settled refugees under health and demographic surveillance in the world (12).

Our analysis is based on 21 villages covered by the AHDSS from 1993–2009 and uses data from women aged 15–49 who gave birth in Agincourt.^1^ Information on births, including limited information on the use of contraception prior to a birth, is collected in regular census rounds conducted since the baseline in 1992. Additional data on individuals and households are collected through special modules in the annual census update. The education of individuals is updated regularly and our analysis uses the highest level of education recorded for women. Women's employment status was captured in 2000, 2004, and 2008. Household asset status has been measured every second year since 2001 and is used to create measures of household wealth.^2^

The standard method for estimating the age pattern of fertility (technically referred to as ASFR) and the level of fertility measured by the TFR are used to examine fertility trends. The latter is defined as the average number of children that a woman would have by the end of her reproductive life if the current age pattern of fertility were to remain unchanged. Descriptive statistics are used to describe changes in the age pattern of fertility over time. A discrete time event framework is used to evaluate women's progression from a first to a second birth within five years and smoothed survival curves are presented. Other socio-economic trends are examined by estimating levels of employment, household wealth, and formal education.

## Results

### Total fertility rates

**Fig. 1:**
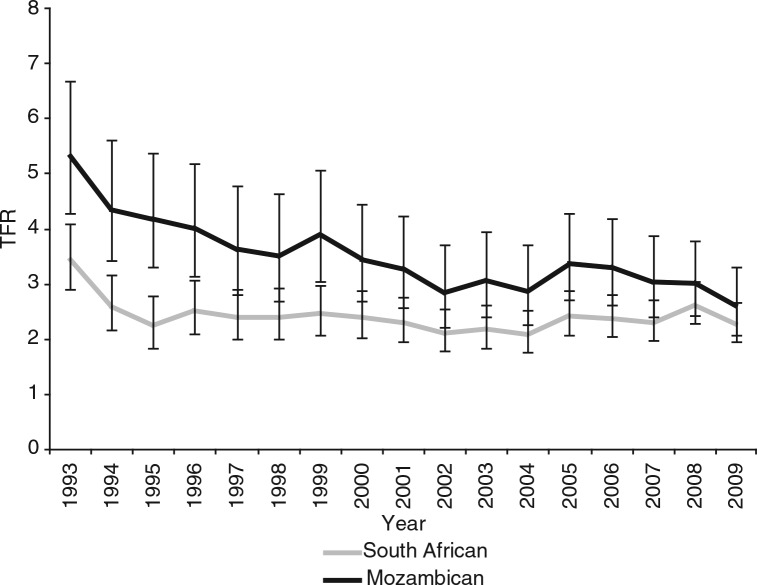
Total fertility rates (TFR) of South Africans and Mozambicans in Agincourt 1993–2009 with a 95% confidence interval.

Figure 1 is based on prospective data beginning in 1993 which shows fertility declining significantly in both population groups in the early 1990s. Figure 1 also shows that Mozambican refugees had higher fertility rates than South Africans until late 2000s, 20–29 years after their initial influx.

Fertility levels were quite different in the two populations during the 1990s, with Mozambican women maintaining higher fertility than South Africans. Thereafter, the two populations increasingly exhibit similar fertility levels, converging from 2000 when the confidence intervals around the fertility estimates for the two groups started overlapping. Figure 1 also suggests a stall in the fertility decline of both populations since 2002. This corroborates research suggesting that fertility decline may have stalled in South Africa (6, 15).

### Patterns of childbearing

The convergence of total fertility of the two population groups is driven primarily by the decline in fertility among Mozambican women to the levels of South African women. This suggests that Mozambican women were adopting fertility behaviours similar to those of the host population. To test this hypothesis we compared age-specific fertility rates and the timing of first and second births between the two populations at the beginning and end of the observation period.

#### Age-specific fertility rates

**Fig. 2:**
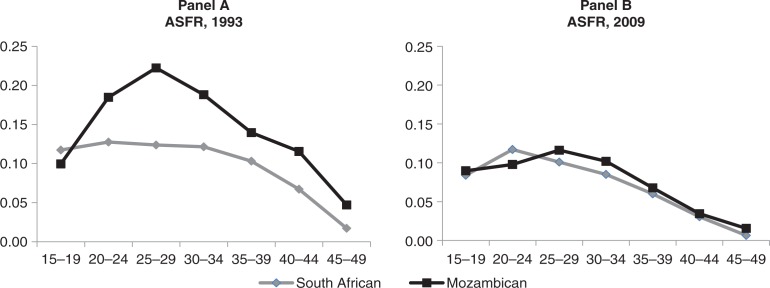
Age specific fertility rates (ASFR) of the two population groups in the AHDSS, 1993 and 2009.

Figure 2 compares the age-specific fertility rates of Mozambican and South African women in 1993 and 2009. Panel A of Fig. 2 demonstrates a strikingly different age pattern of childbearing in 1993 between the two groups of women: Mozambicans have slightly lower adolescent fertility rates (aged 15–19) but higher fertility rates in all other age groups, with a significant peak at ages 25–29. In contrast, South Africans have fairly constant fertility rates across women aged 15–34 until they begin to fall and continue a downward trend at older ages.

However, by 2009, Panel B of Fig. 2 shows that the age-specific fertility patterns for Mozambican and South African women were quite similar, with very little difference at all ages. The gap between the age-specific fertility rates of the two groups found in 1993 disappears due to significantly lower fertility rates for Mozambican women at ages 20–49. Fig. 2 also shows that fertility decline in the Mozambican population has come most notably from declines in fertility across ages 20–34.

#### Timing of first births

The age-specific fertility rates suggest similarly high levels of adolescent fertility for Mozambican and South African women. Further analysis of the age distribution of first births for Mozambican women (Table 1) shows increases in the proportion of first births to adolescents over time. This suggests that Mozambicans are following a pattern found in the area by previous research (16) of consistently high adolescent fertility despite a decline in overall fertility.

**Table 1:** Age at first birth by nationality and period (%)

	1993–1995		2003–2005	
		
	Mozambican	South African	Mozambican	South African
Age at first birth				
15–19	56.37	60.53	59.27	52.57**
20–24	29.78	29.51	28.38	32.75+
25–29	9.74	7.29+	10.04	10.12
30–40	4.12	2.67	2.32	4.56*
*N*	534	1,125	518	1,383

Significance test for difference between Mozambican and South African.

+ Significant at 0.1 level.

* Significant at 0.05 level.

** Significant at 0.01 level.

Table 1 also shows that more recently in the period 2003–2005, Mozambican women have a statistically significant higher percentage of first births occurring to adolescents (59.3%) than South African women (52.6%). For that same period, the average age at first birth for Mozambican women is below 20 (19.7) and above 20 (20.36) for South African women. While adolescent fertility appears to be decreasing for South African women, it appears to be increasing for Mozambican women.

Further analysis also suggests lower contraceptive use by Mozambican women prior to their first birth. At the time of their first birth, Mozambican women consistently reported lower contraceptive use prior to conceiving than South African women. Five per cent of Mozambican women compared to 9.5% of South African women with first births from 1995 to 1999 reported using contraception at some time before their first birth. These figures were 23% and 28%, respectively, for first births occurring from 2003 to 2005. High adolescent fertility has been a source of concern in South Africa and so it is important to recognise the lower use of contraception before a first birth as well as the increase in the percentage of first births to adolescents for Mozambican women (16).

#### Timing of second births

**Fig. 3:**
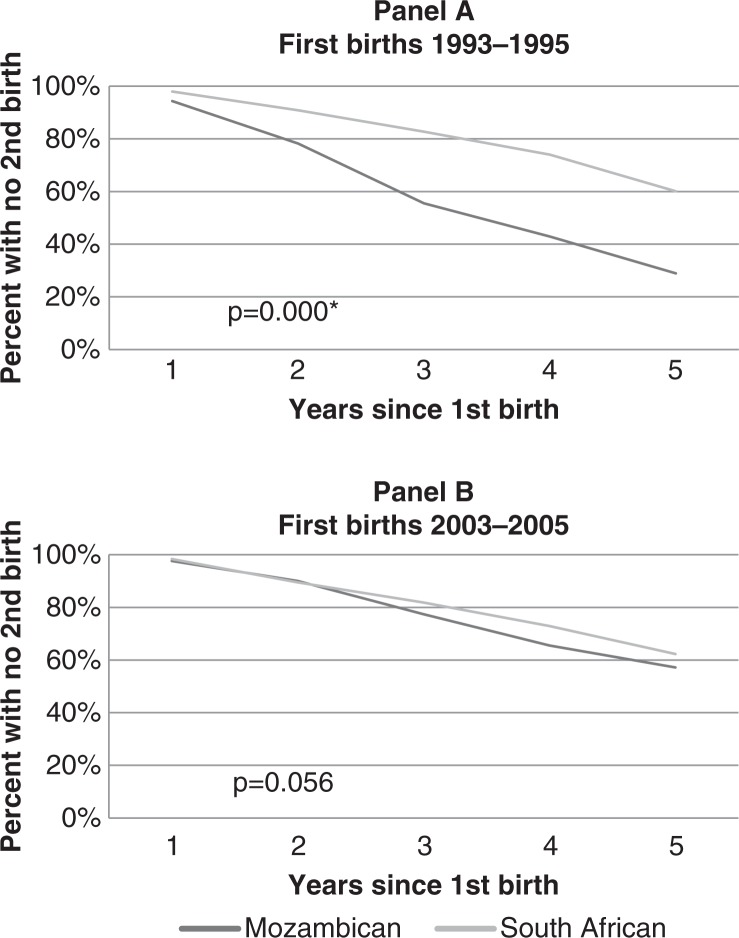
Smoothed discrete survival function curves showing the percentage of women with no second births up to 5 years after a first birth in two time periods 1993–1995 and 2003–2005 by nationality. *The curves are statistically significant at the *p* < 0.001 level according to a log-rank test for equality of survivor functions.

Previous research on the fertility of host South Africans has shown that fertility decline for African South Africans has been driven by significant widening of birth intervals explained primarily by increases in the use of modern contraception (17). Wide birth intervals may also be a result of adolescent non-marital fertility followed by late marriage and low marital fertility (16). Contraceptive use in Agincourt has been shown through qualitative research to be used primarily after the first birth to delay subsequent births (16, 18, 19). In the early 1990s, a majority of South African women in Agincourt delayed second births for more than five years, while a majority of Mozambican women did not. Panel A of Fig. 3 shows that only 40% of South African women that had first births between 1993 and 1995 had a second birth within five years as compared to more than 70% of Mozambican women. Over time, however, the pattern of second births among Mozambican women has become similar to that of South African women. Panel B of Fig. 3, shows that for women experiencing a first birth between 2003–2005 the percentage of Mozambican and South African women progressing to a second birth within five years was similar, relatively low, and not statistically significant (43% and 38% respectively).

The changes in age-specific fertility rates, timing of first births and extended first birth intervals indicate that Mozambican women are achieving lower fertility by adopting patterns of childbearing typical for South African women in Agincourt.

### Trends of selected socio-economic characteristics

**Fig. 4:**
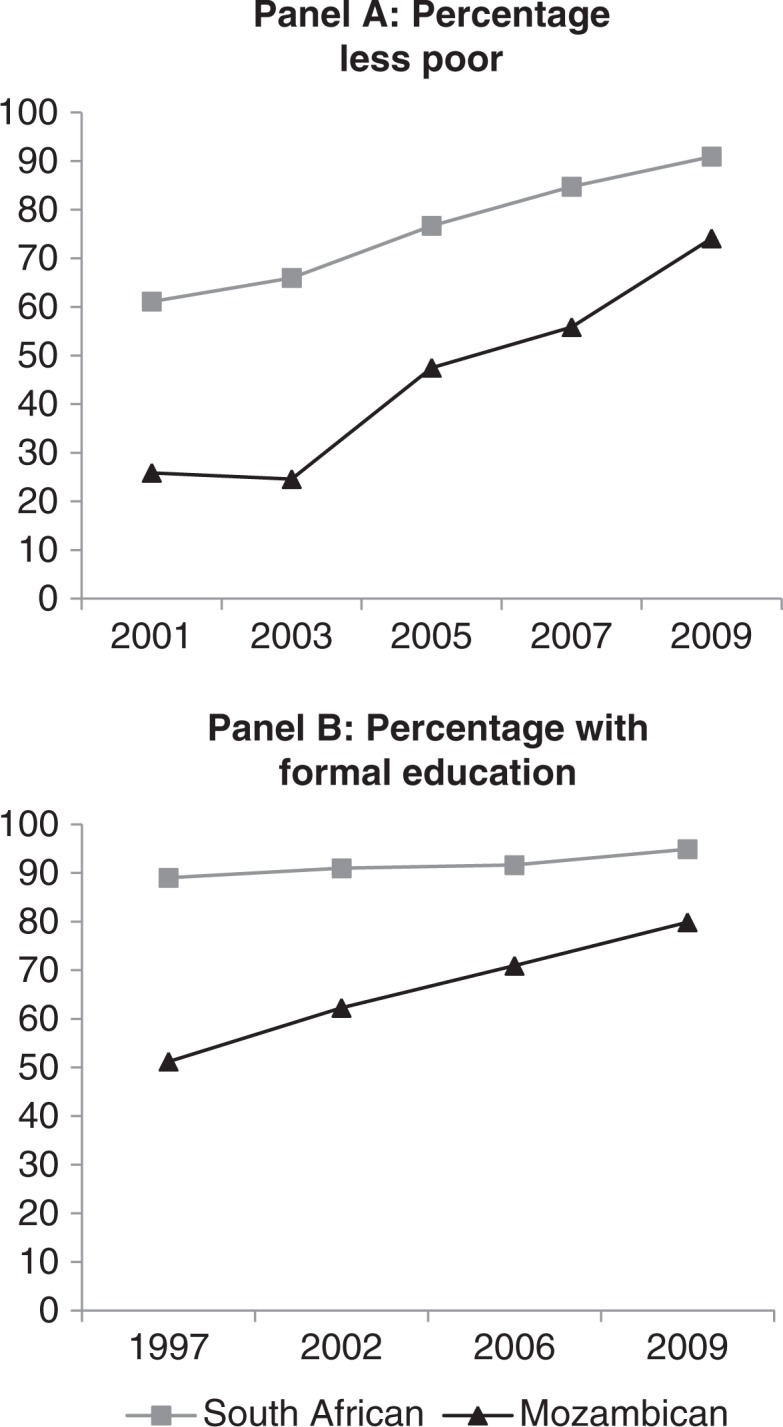
Household wealth status 2001–2009 and education 1997–2009 of South African and Mozambican women of age 15–49 in the AHDSS.

To further explore the fertility decline and the convergence of fertility in the two populations, we examine select socio-economic factors that may be ‘underlying’ drivers of the decline in the TFR among the Mozambicans. Increases in education, labour force participation and income have been found to reduce fertility (20–22). Historically, Mozambicans have been socially and economically disadvantaged in the Agincourt sub-district. However, over time their socio-economic status has improved and policy changes in 2004 enabled Mozambicans (as permanent residents) to access South African state resources such as child grants and old-age pensions (23). Panel A of Fig. 4 shows the percentage of women of childbearing age whose households fall into the category ‘less poor’, or the top socio-economic strata of the wealth index. This demonstrates the economic gains of Mozambicans and their convergence with South Africans over time.

Panel B of Fig. 4 shows a rapid increase in the percentage of Mozambican women with some formal education and a slight increase for South African women. Increased access to formal education likely contributed to the decrease in fertility for Mozambican women.

An analysis of the labour force participation of women of reproductive ages shows that formal employment increased slightly during the past decade for South Africans (from about 28% in 2000 to 30% in 2008) but decreased for Mozambicans (from about 27% to 23% over the same period). The very high unemployment of both groups suggests limited formal economic opportunities for women, which might have contributed to the recently observed stall in fertility decline.

Education and wealth indicators suggest that over the period of study Mozambican women's status improved and converged with that of South African women. However, these gains are relatively modest and Mozambican women remain disadvantaged, particularly in relation to formal employment, within the relatively poor population of the rural setting.

## Discussion and conclusion

Approximately 20 years after the civil war in Mozambique, demographic characteristics of self-settled refugees of Mozambican origin in Agincourt are converging with those of their South African hosts. While the TFR in Mozambique itself has remained near 5 (24), the Mozambican TFR in Agincourt was 2.6 in 2009, its lowest level to date. Both population groups now show similar fertility patterns, with a high proportion of first births in the 15–19 age range and delayed childbearing thereafter.

The findings of this study suggest adaptation of the Mozambican refugees in the AHDSS to the fertility patterns of their host community. Adaptation theory states that exposure to cultural norms and local costs of childbearing will lead migrants to change their fertility behaviour to converge with that of natives in the destination (25). This appears to be the case, particularly through 2005 when the majority of the population of Mozambican women in Agincourt were former refugees. The fertility of more recent Mozambican migrants might additionally be suppressed due to the disruption caused by migration.

The adaptation of Mozambican refugees to the lower fertility regime in South Africa has important implications for many areas of sub-Saharan Africa hosting refugee populations. The adaptation of Mozambicans in South Africa is likely facilitated by a shared language and culture. Self-settled refugees are also probably more likely to be exposed to and adjust to the local norms of childbearing compared to refugees living in camps.

Access to contraception through the South African health system is a key component of the decrease in fertility of Mozambicans. Another important component is the improvement in socio-economic status partly attributable to access to education and host government social grants. Reducing the economic disadvantage of refugees and integrating refugees into local programmes and services encourages adaptation and can compensate for other factors that may otherwise increase the fertility of refugees such as poverty, lack of education, and lack of reproductive health services. Integration encourages adaptation and will likely benefit host communities by lowering the fertility of refugees.

Overall fertility decline in Agincourt over the past few decades has been driven primarily by the decline in fertility of Mozambican women. South African women's total fertility declined primarily in the early 1990s and has been wavering around 2.5 since 1995. Fertility decline has also been minimal for Mozambican women since 2002. With fertility decline stalling in both groups it remains to be seen if fertility will go below replacement level (2.5 in South Africa) as predicted by earlier research (26). Further research is needed to determine the impact of factors such as infant mortality, changing marriage patterns, migration, and HIV on fertility in Agincourt and throughout South Africa.

Findings presented here suggest a few areas of future intervention that would be helpful in settings such as Agincourt. The pattern of childbearing in Agincourt shows that delaying first births could reduce overall fertility rates. Others have argued that family planning programmes in South Africa need to be reoriented to address the contraceptive needs of adolescents before first births (18). Since contraception and family planning advice are provided largely by nurses working from primary health care facilities, strengthening the adolescentfriendly and responsiveness of clinic-based services is important. Programmes in Agincourt should pay special attention to Mozambican adolescents, whose reported contraceptive use is lower than that of South Africans. Increasing contraceptive use before age 20 will lower adolescent fertility and overall fertility rates. Furthermore, if programmes can successfully increase condom use, they may have the added benefit of reducing HIV transmission.

In other settings, increasing access to family planning and reproductive health programmes for all women has been shown to improve women's economic and health outcomes and to enhance economic growth (27). However, the lingering effects of apartheid policies of differential development are evident in the low education and very high unemployment of women in Agincourt. Programmes that improve education and create job opportunities for all women, particularly Mozambican women, are needed to complement improvements in family planning and reproductive services in order to overcome endemic poverty in the area. Efforts to improve reproductive health services and improve the socio-economic status of women are likely to be synergistic, with each encouraging lower fertility and economic growth.

The primary limitations of our study are data driven. We do not have information on important variables such as prospective data on marriage, fertility desires, or detailed information on contraceptive use, to run models examining the proximate determinants of fertility.

## Footnotes

1 The proportion of Mozambican women in the population remained about one-third throughout the study period. Since 1994 some Mozambicans have migrated voluntarily into the area and are included in the analysis with Mozambican refugees. However the percentage of in-migrants was minimal for the majority of the study period. Until 2007 less than 4% of the Mozambican population in the AHDSS were in-migrants. By 2009, 17.6% of the Mozambican population in the AHDSS were in-migrants.

## Footnotes

2 Every second year starting in 2001, an asset survey has been conducted in all households of the Agincourt surveillance site. A set of these household assets can be used to develop a wealth (SEP) index for each household (13). The sum of these assets was used to create an absolute asset count for each household. This score was then used to define three socio-economic strata based on tercile cut-offs and categorised: more poor, poor, less poor (14).

## Footnotes

‡ There are family and kinship ties between present-day South Africans and Mozambicans that extend back for generations. Many thousands of Mozambicans entered South Africa as refugees during and after the civil war during the 1970s and into the 1980s. Most who settled in the vicinity of Agincourt sub-district chose to remain and this was facilitated by common kinship and family ties. Today, a high proportion of these former Mozambican refugees are eligible for and have accessed South African ID documents and enjoy all the rights of South African citizenship. Their children, born in South Africa, are regarded as fully South African.
